# Cardiac Metastasis in a Living Patient With Laryngeal Squamous Cell Carcinoma

**DOI:** 10.7759/cureus.16214

**Published:** 2021-07-06

**Authors:** Polina Tregubenko, Sherrie White, Valeriy Zvonarev, Abhishek Kumar

**Affiliations:** 1 Internal Medicine, Jacobi Medical Center/Albert Einstein College of Medicine, Bronx, USA; 2 Pathology, Jacobi Medical Center/Albert Einstein College of Medicine, Bronx, USA; 3 Psychiatry, University of Missouri Kansas City School of Medicine, Kansas City, USA; 4 Oncology/Hematology, Jacobi Medical Center/Albert Einstein College of Medicine, Bronx, USA

**Keywords:** cardiac metastasis, fibrinous pericarditis, head and neck cancer, laryngeal carcinoma, radiation, squamous cell carcinoma

## Abstract

Metastatic cardiac tumors are mainly diagnosed postmortem, while cardiac metastases of laryngeal cancer are exceedingly rare. We report a case of laryngeal carcinoma with subsequent metastatic disease to the heart diagnosed nine months after surgical resection of laryngeal cancer. Additionally, we attempted to* *summarize published case reports of laryngeal cancer with cardiac metastasis. Retrospective chart review and literature search via PubMed and Google Scholar were performed. Twenty cases of laryngeal squamous cell carcinoma (SCC) with cardiac metastatic tumors were identified. We described demographics, typical features seen on diagnostic studies, and analyzed current literature on incidence, diagnostic studies, treatment options, and prognosis of secondary cardiac tumors. More data are needed to decide on the optimal treatment strategy for metastatic cardiac disease.

## Introduction

Cardiac involvement by primary and secondary tumors is one of the least investigated oncology subjects [1]. Tumors involving the heart and pericardium are far more likely to be secondary (metastatic) than primary, occurring in up to 18.3% of patients with metastatic disease [2]. Cardiac metastasis of laryngeal cancer is a rare condition, with one of the earliest reports of infiltrative cardiomyopathy secondary to laryngeal carcinoma written by Linell et al. in 1922 [3], and only several cases reported in the literature over the past century. We report a case of laryngeal squamous cell carcinoma (SCC) with subsequent metastatic disease to the heart.

## Case presentation

**Figure 1:**
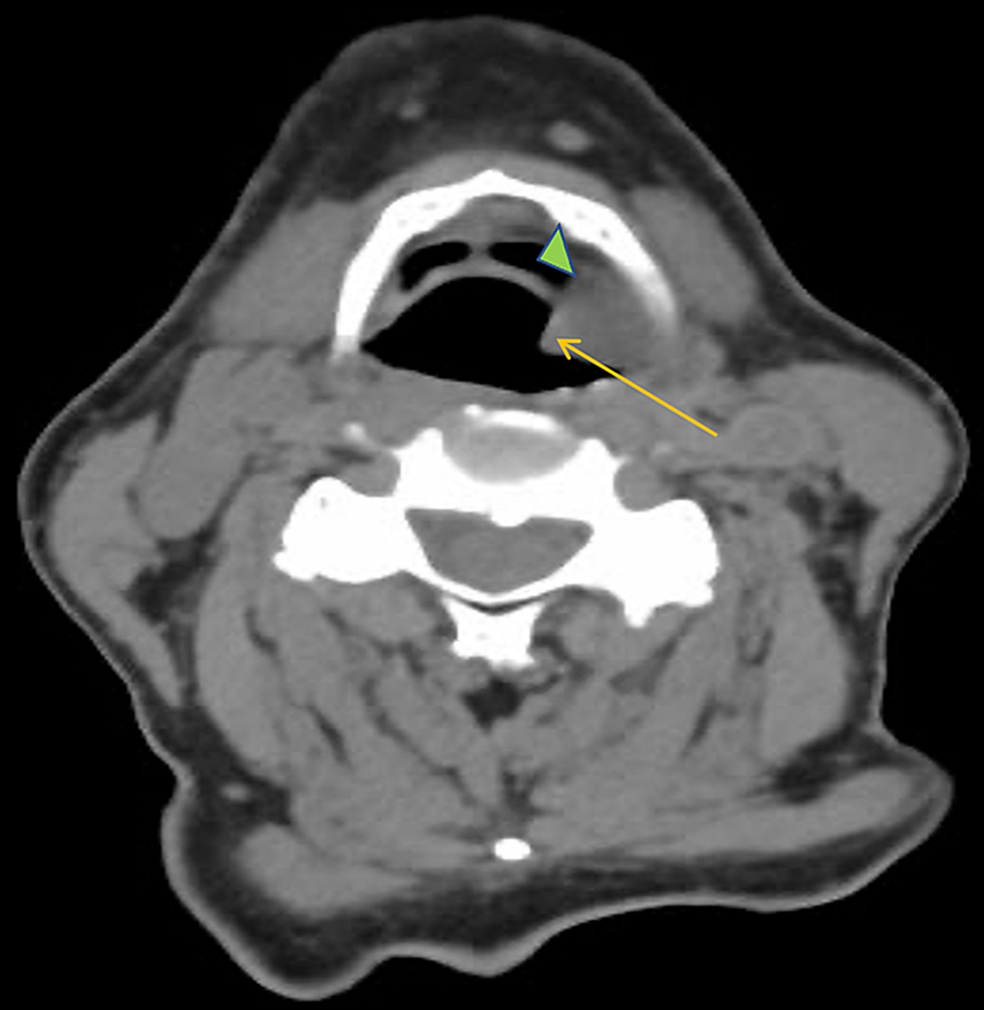
CT scan of the neck showed a soft tissue mass (arrow) on the left piriform sinus and vallecula (arrowhead) extending inferiorly to the false vocal cords (out of view).

A 63-year-old male with a history of hypertension and chronic obstructive pulmonary disease presented with hoarseness and weight loss of 40 pounds over seven months. He had a 15-pack-year smoking history. Direct laryngoscopy revealed a fungating mass on the left vocal cord. Computed tomography (CT) of the neck showed a soft tissue mass on the left piriform sinus and vallecula extending inferiorly to the false vocal cords, measuring 2.7 cm in maximal dimension (Figure 1). The mass was biopsied, and the patient was diagnosed with invasive moderately differentiated keratinizing SCC of the larynx (pT3N2bM0), for which he underwent total laryngectomy with radical lymph node dissection and left hemithyroidectomy.

**Figure 2:**
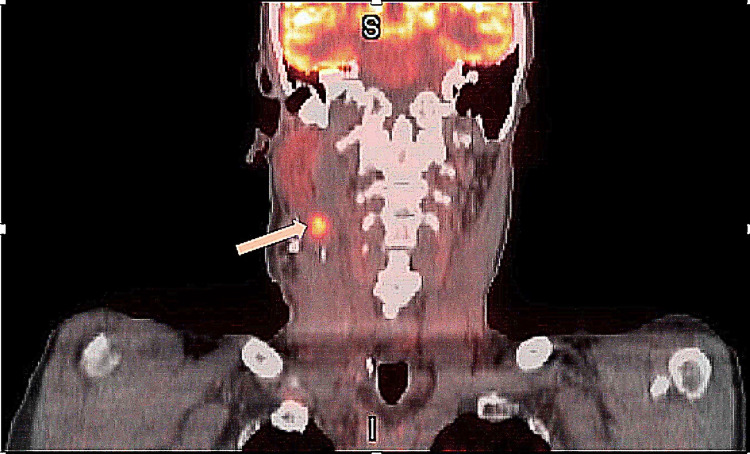
Coronal view PET scan showing increased fluorodeoxyglucose (FDG) uptake in the right level 2B (upper jugular) lymph nodes (arrow).

**Figure 3:**
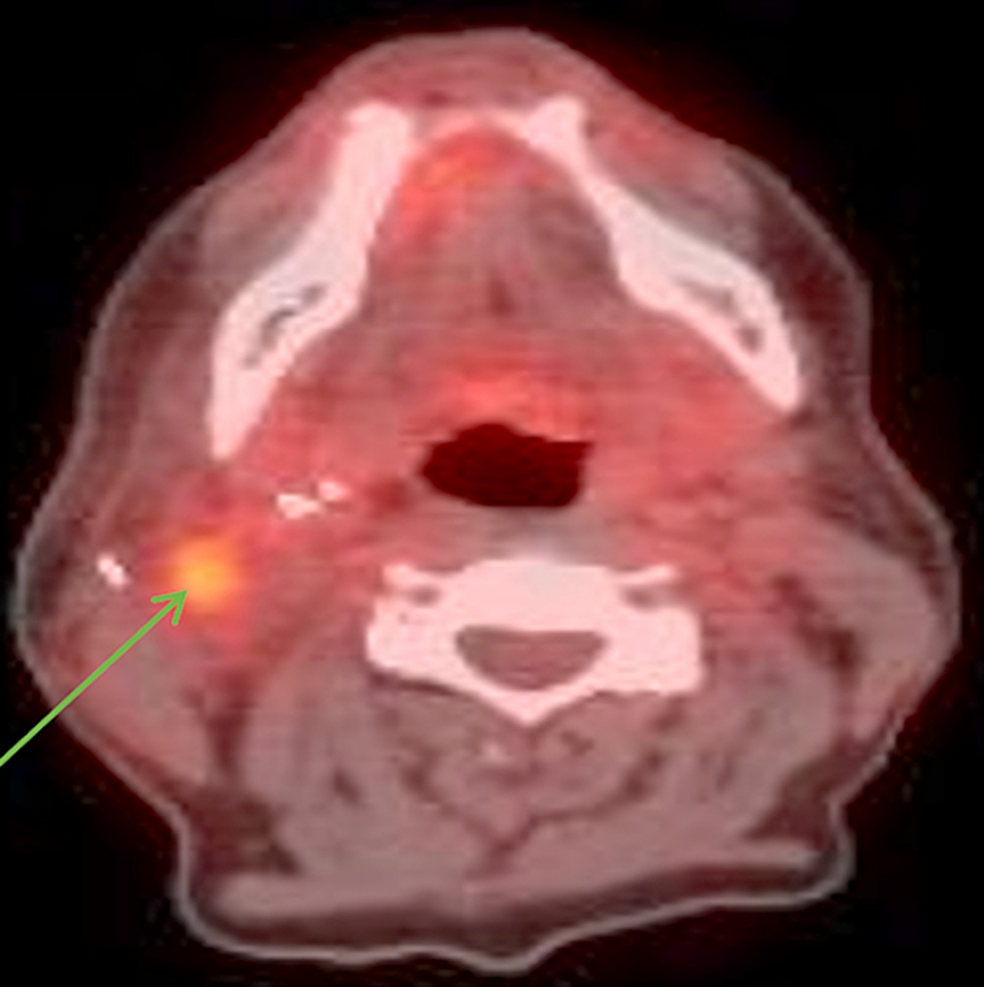
Axial view PET scan showing right-sided level 2B lymph nodes (arrow) with increased FDG uptake, suspicious for metastatic disease.

Positron emission tomography (PET) scan obtained three months after the surgery showed increased fluorodeoxyglucose (FDG) uptake in the right level 2B lymph nodes suspicious for metastasis and an ill-defined focus in the left supraclavicular fossa (Figures 2, 3).

**Figure 4:**
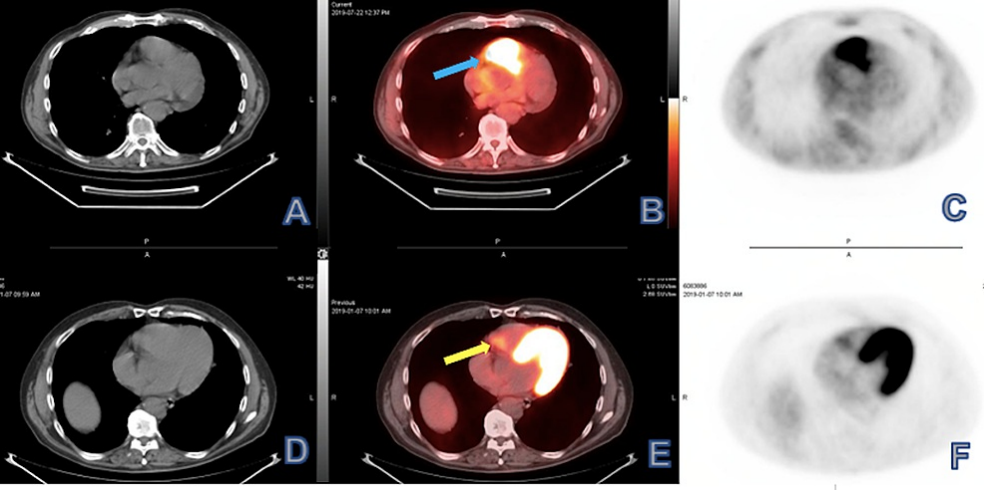
Restaging PET scan axial view (A-C) demonstrated increased focal uptake correlating to pericardial region of the right heart (B, arrow), which appeared as small nonspecific focus (E, arrow) on initial study (D-F).

The patient was treated with concurrent chemoradiation with a cumulative total radiation dose of 69.96 Gy, with weekly cisplatin for seven weeks. The restaging PET scan three months after completion of the concurrent chemoradiation showed no residual disease in the neck; however, it revealed a new hypermetabolic focus correlating to the right heart chamber suspicious for a pericardial mass (Figure 4).

**Figure 5:**
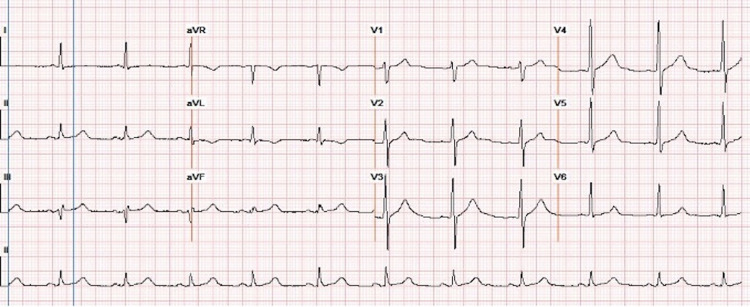
Baseline ECG showing no abnormalities

**Figure 6:**
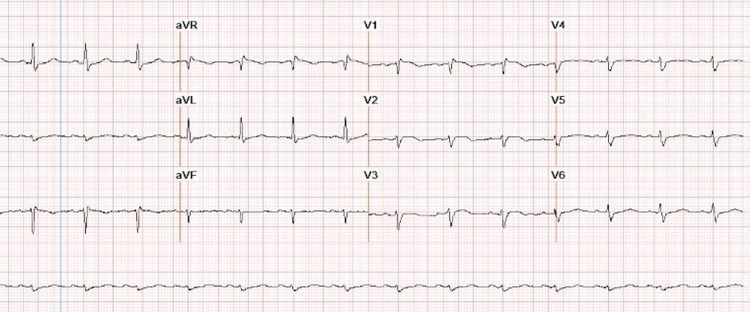
ECG after diagnosed cardiac metastasis showing lower voltage and incomplete right bundle branch block

In comparison with baseline electrocardiogram (ECG), lower voltage and new incomplete right bundle branch block were noted (Figures 5, 6).

**Figure 7:**
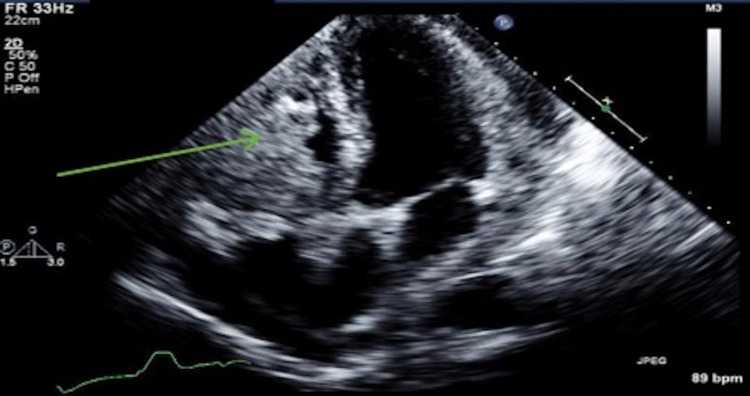
Patient's transthoracic echocardiogram with a large soft-tissue mass (arrow) involving the right ventricle (RV) wall and cavity from the tricuspid annulus to the RV outflow tract. Maximal dimension recorded was 4.6 cm x 3.6 cm.

Transthoracic echocardiography (TTE) revealed a large soft tissue mass measuring 4.9 cm x 2.7 cm in the basal-mid right ventricle (RV) adjacent to the wall and thickened pericardium associated with RV dilation and pericardial effusion (Figure 7).

**Figure 8:**
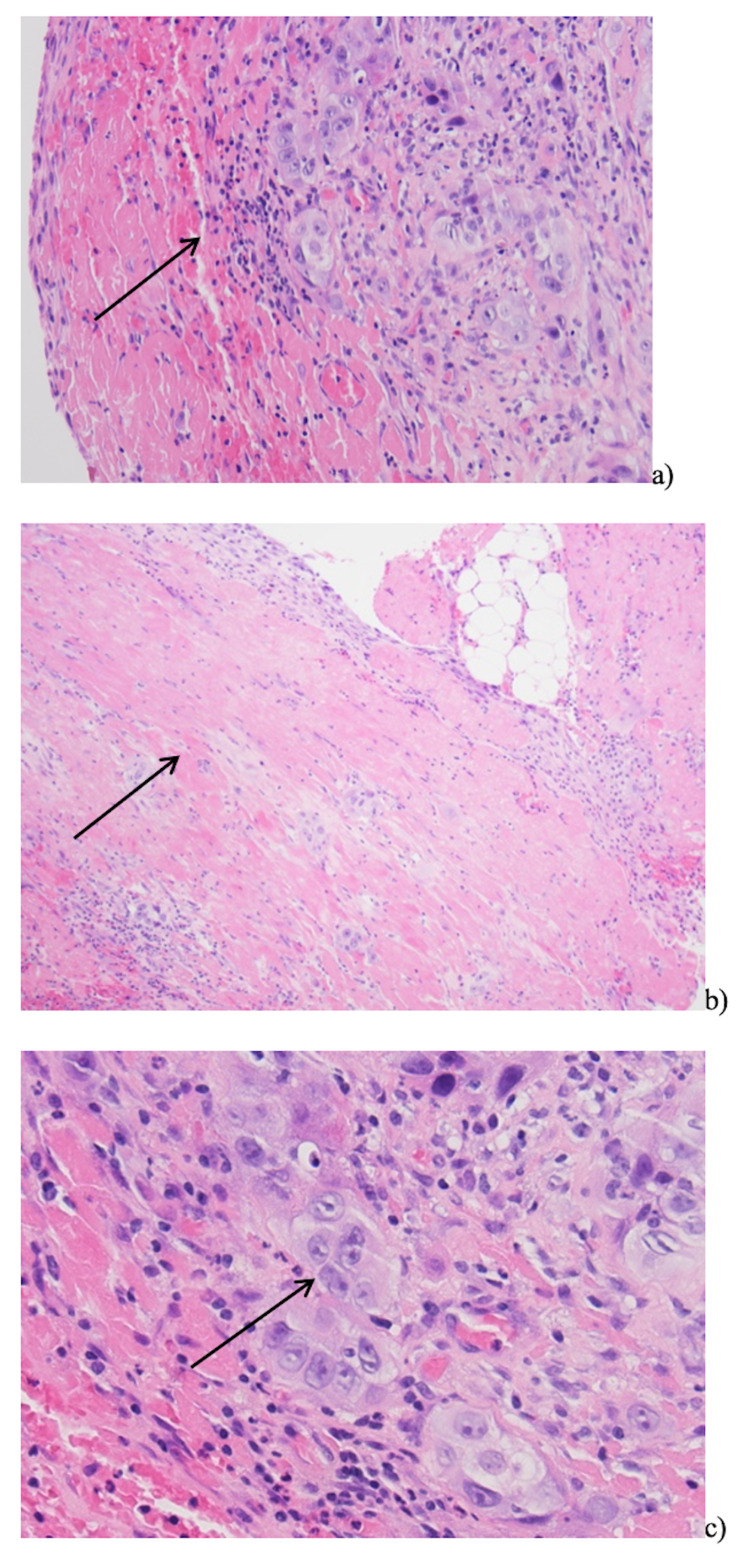
Pathological findings on pericardial biopsy consistent with metastatic squamous cell carcinoma. (a) acute fibrinous pericarditis with inflammatory infiltrate (arrow); (b) organizing fibrinous pericarditis (arrow); (c) atypical cells (arrow) in the background of fibrin, neutrophils, lymphocytes, and reactive mesothelial cells.

The mass increased in size on repeat study one week later. Pericardial window with pericardial biopsy was performed and showed atypical cells in pericardial debris, neutrophils, lymphocytes, and reactive mesothelial cells suspicious for metastatic SCC (Figure 8).

The patient was admitted for bilateral pulmonary embolism, acute deep vein thrombosis, and increased size of the RV mass. Due to poor performance status and rapid clinical decline, the patient was not considered a chemotherapy candidate. Treatment with palliative radiotherapy (radiation dose of 20 Gy in five fractions) to the heart was initiated in an attempt to achieve regression of cardiac lesion; however, the patient expired shortly after his second session of radiotherapy.

## Discussion

The incidence of primary and secondary cardiac tumors has been primarily reported in postmortem studies, and although primary tumors are extremely uncommon, with reported rates of 0.001% to 0.28% [2], the incidence of secondary tumors varies significantly, ranging from 1.23% to 18.3% [4,5]. Lung and breast cancer account for most neoplasms metastasizing to the heart due to their high prevalence [6]. Tumors that are particularly prone to cardiac metastases include melanomas, sarcomas, hematologic malignancies, germ cell tumors [4], epidermoid lung carcinoma, urinary tract tumors [7,8], and malignant thymoma [9].

Lymphatic propagation is the primary mode of cardiac metastases dissemination [2], although hematogenous metastasis and direct extension have also been reported [6]. The structure of the lymphatic system in the heart may explain the relatively low incidence of secondary tumors in the heart compared with other organs [2].

Pericardium accounts for 52% of metastases (including 33% in the epicardium), 42% are located in the myocardium, and only 6% affect the endocardium [10]. Usually, hematogenous dissemination leads to endocardial involvement, whereas metastases to the myocardium or epicardium almost exclusively result from retrograde lymphatic spread through tracheal and broncho-mediastinal lymph trunks [2].

Patients with cardiac metastasis rarely develop specific symptoms, and most of the cases are diagnosed on autopsy. Pericardial metastases can provoke cardiac tamponade or constriction and are the most commonly diagnosed [6]. Intracardiac metastases are typically found within the right-sided chambers of the heart [9]. Intracavitary tumors are rare, and they can cause valve stenosis or regurgitation through valve impingement or embolization to the pulmonary or systemic circulation, causing infarction and distant metastases [6].

Table 1 summarizes all the published reports of cases of laryngeal SCC with cardiac metastasis.

**Table 1:** Cases of intracardiac metastasis of laryngeal cancer Abbreviations: AFib – atrial fibrillation; AV – atrioventricular; CHF – congestive heart failure; CRT – chemoradiation; CT – computed tomography; CXR – chest radiograph; ECG – electrocardiogram; Echo – echocardiogram; F – female, HSM – holosystolic murmur; HR – heart rate; IVS – interventricular septum; L – left; LV – left ventricle; M – male; Mo(s) – month(s); MRI – magnetic resonance imaging; N/A – not available; PET – positron emission tomography; PVC – premature ventricular contraction; R – right; RBBB – right bundle branch block; RT – radiotherapy; RV – right ventricle, RA – left atrium; RLE – right lower extremity; RT – radiotherapy; RWP – R wave progression; SCC – squamous cell carcinoma; SEM – systolic ejection murmur; SOB – shortness of breath; VT – ventricular tachycardia * short decrescendo diastolic murmur at left sternal border 3rd interspace that increased in intensity with inspiration **distended jugular veins, hepatomegaly, bilateral pitting oedema

Author (year), country	Age, gender	Primary site	Stage	Primary treatment	Time from Dx to cardiac mets	Symptom/physical exam findings	Diagnostic test	ECG changes	Cardiac metastasis location	Metastasis treatment
Linell (1922), UK [3]	43 y/o, F	Larynx	IV	Tracheotomy (inoperable tumor)	12 days	Severe pain over the precordium	Autopsy	N/A	Pericardium, R auricle	None
Palmer (1964), USA [11]	59 y/o, M	R piriform fossa, aryepiglottic fold, R hypopharynx	N/A	Surgery	1 mo.	Confusion, shock	Autopsy	HR of 180 with bigeminy, tachycardia	Myocardium	None
	49 y/o, M	L piriform fossa	N/A	Surgery	13 mos.	None	Autopsy	N/A	Pericardium, myocardium	None
	58 y/o, M	L piriform fossa	N/A	Surgery	11 mos.	None	Autopsy	N/A	Myocardium	None
	63 y/o, M	L piriform fossa	N/A	Surgery	12 days	Tachycardia, intractable CHF	Presumptive by CXR and EKG; autopsy	Nonspecific changes in the T-waves	Myocardium, pericardium	None
Harrer (1970), USA [12]	69 y/o, M	Larynx	N/A	N/A	2 mos. (to death)	None	Autopsy	N/A	Intraventricular septum	None
	54 y/o, M	Larynx	N/A	N/A	26 mos. (to death)	None	Autopsy	N/A	Epicardium	None
	32 y/o, M	Larynx	N/A	N/A	4 mos. (to death)	None	Autopsy	N/A	Pericardium	None
	63 y/o, M	Larynx (anterior commissure, L vocal cord, the base of the epiglottis)	II	RT + surgery (negative surgical margins)	1 year (15 mos. to death)	Chest pain L anterior side; PVC; grade 3 SEM, grade 2 diastolic murmur*	Autopsy	First degree heart block, RBBB, pathologic Q-waves	Pericardium, epicardium, myocardium of LV, RV, RA, endocardium	None
Barton (1979), UK [13]	58 y/o, M	Epiglottis, R aryepiglottic fold, arytenoid and false vocal cord	N/A	Surgery + RT	17 mos.	Chest infection, haemoptysis	Autopsy	N/A	Pericardium, LV, LA, RV	None
Tallon (1990), Canada [14]	50 y/o, F	Hypopharynx, lateral pharyngeal walls, posterior cricoid, larynx	T3N2M0	Tracheostomy, RT	8 mos.	SOB, fatigue, anorexia, productive cough	Autopsy	Sinus tachycardia, poor RWP, nonspecific S-T changes in inferior leads	RV endocardium, tricuspid valve	None
Larkin (1994), USA [15]	41 y/o, M	Larynx	IV	Surgery	N/A	Syncope, irregular HR	Echo; autopsy	Rapid AFib; RBBB, persistent ST-elevation in V1 to V6	Pericardium, RV	None
Renders (2005), Belgium [6]	54 y/o, M	Larynx	T4N2M0	RT	4 mos.	SOB, fatigue, atypical chest discomfort; signs of RV failure**, bilateral basal crackles, HSM over tricuspid area	Echo, RV angiogram, CT, MRI, percutaneous RV biopsy	Low QRS voltages, ST segment elevation in leads V2 to V5, 3^rd^ degree AV block	RV	Compassionate care
Gullulu (2006), Turkey [1]	63 y/o, M	Larynx	N/A	CRT + surgery	1 year	Dull sternal pain, nausea, diaphoresis; coarse crackles in the lower right lung	Postmortem biopsy	New convex ST segment elevation in V1-V4 without Q waves	Myocardium	None
Alhakeem (2008), USA [9]	49 y/o, M	Larynx	N/A	Surgery + RT	2 years	Dyspnea, left-sided chest pain, hypotension, tachycardia	Echo, CT	Inverted T waves in the infero-lateral leads	RV free wall, LA, LV lateral free wall, IVS, annulus of the tricuspid valve	N/A
Kavanagh (2012), Croatia [16]	50 y/o, M	R hemilarynx, sublingual carcinoma	N/A	RT + surgery (negative surgical margins)	5 years	Dyspnea; cyanosis; sudden cardiac arrest	Autopsy	Ventricular fibrillation, asystole	RV, conduction system infiltration	None
Rangel (2012), Portugal [8]	71 y/o, M	Larynx	N/A	Surgery + RT	1 year	Atypical chest pain, worsening fatigue	Echo, CT	Sinus tachycardia, poor RWP, slight ST segment elevation in leads V1-V3	RV apex and free wall, IVS; pericardium adjacent to LV lateral wall	Palliative treatment proposed
Gunduz (2015), Turkey [17]	47 y/o, M	Larynx	N/A	N/A	N/A	Dyspnea	Echo	N/A	LV endo-myopericardium; RV pericardium	None
Vaduganathan (2016), USA [18]	69 y/o, M	Larynx	N/A	N/A	N/A	Fatigue, stable VT requiring cardioversion	Echo, cardiac MRI	ST elevations in I, aVL, V2-V4, V6; 2:1 AV block	LV mass involving apico-anterolateral and apical septal myocardium	Systemic chemotherapy
Tregubenko (2021), USA (current study)	63 y/o, M	Larynx	T3N2bM0	Surgery + CRT	6 mos.	None	PET, echo	Poor RWP, nonspecific ST-T changes in inferior and precordial leads	RV	Palliative RT

Three patients from Burke and Herbut autopsy series were not included in our analysis due to a lack of data [19,20]. There is a significant predominance of male patients (90%), with ages ranging from 32 to 71 years old, and more commonly people affected by laryngeal cancer in their sixth and seventh decade of life. The larynx was the primary site in all cases except one, where a patient with sublingual and laryngeal carcinoma developed metastases to the lung and heart [16]. Primary treatment involved surgery in five cases (25%) [11,15], radiotherapy in one case (5%) [6], combination therapy with surgery and radiation in five cases (25%) [8,9,12,13,16], and a combination of surgery with chemoradiation in two cases (10%) [1], including current case. Two patients had tracheostomy performed for symptom relief [3,14]. Most cases (80%) had cardiac metastases discovered within two years after the initial diagnosis, except one, where metastatic disease to the heart was diagnosed five years later [16]. While the majority (55%) were diagnosed at autopsy or postmortem biopsy, in six cases (30%), the diagnosis was made in living patients based on imaging studies. No ECG changes were reported in almost half of the cases, and in those who had (including our patient), ECG changes were nonspecific. One case described a patient developing hemodynamically stable, monomorphic ventricular tachycardia (VT), which is usually resistant to antiarrhythmic therapy and is challenging to treat as residual disease and tumor necrosis might serve as ongoing foci for recurrent VT activity [18]. More patients had right-sided chambers of the heart involved (50% versus 30% involving left-sided chambers), although exact location was not reported in 40% of cases. Almost none of the patients received treatment directed towards cardiac metastasis, and in two cases where treatment was initiated, the patient succumbed days after its onset.

The most common clinical manifestations of cardiac metastasis result from large pericardial effusion, tachyarrhythmias, atrioventricular block, and congestive heart failure. Obstruction of the superior vena cava can be observed, and in rare cases, arterial embolic events resulting in ischemic bowel or limbs [9]. Dyspnea is common and may result from pleural effusion, valvular obstruction, and cardiac failure [6]. Other symptoms include chest pain, signs of right-sided heart failure, and systemic embolism. In some cases, sudden death occurs due to myocardial rupture, ventricular arrhythmias, and myocardial infarction [6].

Cardiac tumors may cause rhythm disturbances and ECG changes, of which nonspecific ST-segment and T-wave changes are most commonly seen [8]. In a retrospective analysis by Cates et al., 40% of patients with cardiac metastases had new ECG changes suggestive of myocardial ischemia or injury, including diffuse or segmental T-wave inversion ST elevation [21]. Other reported findings include low voltage, conduction abnormalities, and pseudo-infarct patterns [6]. Atrial fibrillation, bundle branch block, and persistent ST elevation are common sequelae of myocardial tumor deposits reported by Larkin et al. [15]. Localized and prolonged ST-segment elevation without Q-waves appears to be a pathognomonic sign of myocardial tumor invasion [22].

Treatment of cardiac metastasis is often directed toward symptom relief, and palliation since cardiac involvement is mostly found in the setting of widespread metastatic disease and late stages. However, if the heart is the only location of metastasis, treatment may be curative when the primary tumor is under control. Surgical excision may be indicated for a single, well-circumscribed metastasis; other treatment options include mechanical coil embolization of the coronary artery supplying the mass, systemic chemotherapy, chemoembolization, and radiotherapy. Massive pericardial effusion is most efficiently treated with a pericardial window, although intrapericardial chemotherapy, sclerosing agents, isotopes, and radiation have been used to control recurrent pericardial effusion with variable success [6].

The overall prognosis of patients with cardiac metastasis is poor, and long-term survival in patients with intramyocardial invasion is usually limited to a few months at most. Patients with intracavitary tumors can have longer survival, occasionally estimating up to several years [6].

In an autopsy series by Harrer and Lewis, out of 1,047 patients dying from various disseminated malignancies, 14.1% were shown to have cardiac metastases. Four patients with primary laryngeal carcinoma were found to have cardiac metastasis, and in none was the cardiac metastasis the immediate cause of death [12].

In another autopsy series study involving 6,653 autopsies, 31 patients were found to have died from laryngeal carcinoma. Twenty-one tumors (68%) originated in the extrinsic larynx (including the piriform fossae), including four (13%) cardiac metastases [11], whereas 10 tumors (32%) originated in the intrinsic larynx, of which none metastasized to the heart. As reported by Kotwall et al., the most common head and neck carcinoma locations that metastasized distantly were originating from the hypopharynx (60%) and the base of the tongue (53%). Distant metastases of laryngeal cancer are seen in 44% of cases in both supraglottic and glottic larynx being the primary site [23].

Burke et al. reported autopsy findings in 186 patients who had died from a malignant lesion, in which the primary growth arose from squamous epithelium. Twenty-three of those had laryngeal carcinoma, including one patient with distant metastases to the heart [19]. In another autopsy report by Herbut and Maisel, out of 17 deaths from laryngeal carcinoma, cardiac involvement was reported in two patients [20].

Variable clinical presentation, nonspecific symptoms, and unpredictable course make the diagnosis of cardiac metastases challenging. TTE and cardiac magnetic resonance (CMR) imaging are considered ideal non-invasive tools for initial diagnosis [16]. It has been reported that in some cases CMR can be superior to PET/CT in myocardial lesion characterization and differentiation from normal myocardium [24]. When compared with echocardiography (both transthoracic and transesophageal), CMR has shown to be superior in the prediction of specific tumor types and their malignant potential [25]. As reported by Giusca et al. in an analysis of 125 CMR examinations of patients with suspected cardiac masses, diagnosis made with CMR was a correspondent with histology in 78%, compared with 51% of cases diagnosed by echocardiography (p=0.03). It was even more significant for benign versus malignant tumor classification (CMR correctly identified malignant tumor in 98% cases, whereas echocardiography provided the diagnosis in 57% cases, p<0.001) [25]. Another study compared CMR with histology inability to distinguish malignant cardiac and para-cardiac mass from benign, where observers were accurate in the prediction of lesion type (area under the curve 0.88 and 0.92 for two experienced observers), with good interobserver variability [26]. The study also showed that certain morphologic CMR features, such as right-sided cardiac location, inhomogeneity of tumor tissue, and presence of pericardial effusion, are independent predictors of malignant cardiac and para-cardiac tumors.

CT for surveillance and screening tool for distant metastases in head and neck cancer is suggested in patients with three or more positive lymph nodes, lymph nodes of 6 cm or larger, positive low jugular lymph nodes, locoregional recurrence, and a secondary tumor [16]. A transesophageal echocardiography-guided transvenous biopsy is a safe technique suggested for obtaining tissue samples for histological diagnosis [6]. Percutaneous or surgical biopsy with pathological tissue examination is required for a definitive diagnosis of cardiac metastasis.

Approximately 13% of cardiac metastases are symptomatic or directly life-threatening [27]. Some authors suggest that any patient who presents to the emergency department with a history of cancer and shortness of breath, and hypotension should be suspected of having neoplastic pericardiac tamponade [14].

Although an early diagnosis of cardiac metastasis is difficult, it is essential to remember that curative treatment could be possible in some instances. The case presented here highlights the rarity and complexity of diagnosing secondary cardiac tumors due to nonspecific symptoms and association with widespread disease. The chronologic ECG changes and FDG accumulation in the myocardium on PET scan can aid in the early detection of cardiac metastasis [28]. CMR can be highly effective in distinguishing benign from malignant tumors; however, pathology remains the gold standard for an accurate tumor diagnosis [29]. More data are needed to decide on the optimal treatment strategy for metastatic cardiac disease.

## Conclusions

Our analysis revealed that male patients were affected in 60% of cases with a median age of 56 years at diagnosis. Cardiac metastases were diagnosed in 80% of patients within two years of primary laryngeal carcinoma diagnosis. Only six cases were diagnosed in living patients, with most metastases found in the right-sided chambers of the heart. Most of the reported changes in electrocardiography were nonspecific, although 25% of patients had ST-segment elevation without Q-waves, suggestive of myocardial tumor invasion. Current data suggest that interval changes in electrocardiography and increased FDG uptake on positron emission tomography may help detect cardiac metastasis. Echocardiography and CMR are highly informative non-invasive tools for initial diagnosis. More study in humans is needed in this area to make sure medical treatments for metastatic cardiac disease are safe and effective.

## References

[1] Gullulu S, Ozdemir B, Senturk T, Baran I, Cordan J, Filiz G (2006). Cardiac metastasis in a laryngeal carcinoma and associated electrocardiographic changes. Otolaryngol Head Neck Surg.

[2] Bussani R, De-Giorgio F, Abbate A, Silvestri F (2007). Cardiac metastases. J Clin Pathol.

[3] Linell EA (1922). An unusual cause of death from cancer. Br Med J.

[4] Lam KY, Dickens P, Chan AC (1993). Tumors of the heart. A 20-year experience with a review of 12,485 consecutive autopsies. Arch Pathol Lab Med.

[5] Hanfling SM (1960). Metastatic cancer to the heart. Review of the literature and report of 127 cases. Circulation.

[6] Renders F, Vanderheyden M, Andries E (2005). Secondary cardiac tumour originating from laryngeal carcinoma: case report and review of the literature. Acta Cardiol.

[7] Silvestri F, Bussani R, Pavletic N, Mannone T (1997). Metastases of the heart and pericardium. G Ital Cardiol.

[8] Rangel I, Gonçalves A, de Sousa C, Macedo F, Maciel MJ (2012). Metastatic tumor of the right ventricle: an unusual location of tumor involvement in laryngeal carcinoma. (Article in Portuguese). Rev Port Cardiol.

[9] Alhakeem M, Arabi A, Arab L, Guerra RA (2008). Unusual sites of metastatic involvement: intracardiac metastasis from laryngeal carcinoma. Eur J Echocardiogr.

[10] Mukai K, Shinkai T, Tominaga K, Shimosato Y (1988). The incidence of secondary tumors of the heart and pericardium: a 10-year study. Jpn J Clin Oncol.

[11] Palmer BW, Reynders M (1964). Cardiac metastases in carcinoma of the laryngopharynx. Arch Otolaryngol.

[12] Harrer WV, Lewis PL (1970). Carcinoma of the larynx with cardiac metastases. Arch Otolaryngol.

[13] Barton RP, Taylor PC (1979). Cardiac metastases from primary carcinoma of the larynx. J Laryngol Otol.

[14] Tallon JM, Montoya DR (1990). Acute cor pulmonale secondary to metastatic tumor to the heart: a case report and literature review. J Emerg Med.

[15] Larkin M, Maycon RZ, Schreiner DT (1994). Cardiac metastasis from laryngeal carcinoma presenting as syncope. Chest.

[16] Kavanagh MM, Janjanin S, Prgomet D (2012). Cardiac metastases and a sudden death as a complication of advanced stage of head and neck squamous cell carcinoma. Coll Antropol.

[17] Gündüz S, Zencirci E (2015). Case images: endomyopericardial metastasis from laryngeal cancer. Turk Kardiyol Dern Ars.

[18] Vaduganathan M, Patel NK, Lubitz SA, Neilan TG, Dudzinski DM (2016). A "malignant" arrhythmia: cardiac metastasis and ventricular tachycardia. Tex Heart Inst J.

[19] Burke E (1937). Metastases in squamous-cell carcinoma. Am J Cancer Res.

[20] Herbut PA, Maisel AL (1942). Secondary tumors of the heart. Arch Path.

[21] Cates CU, Virmani R, Vaughn WK (1986). Electrocardiographic markers of cardiac metastasis. Am Heart J.

[22] Hartman RB, Clark PI, Schulman P (1982). Pronounced and prolonged ST segment elevation: a pathognomonic sign of tumor invasion of the heart. Arch Intern Med.

[23] Kotwall C, Sako K, Razack MS (1987). Metastatic patterns in squamous cell cancer of the head and neck. Am J Surg.

[24] Oda S (2018). Cardiac diffusion-weighted magnetic resonance imaging for assessment of cardiac metastasis. Eur Heart J Cardiovasc Imaging.

[25] Giusca S, Mereles D, Ochs A (2017). Incremental value of cardiac magnetic resonance for the evaluation of cardiac tumors in adults: experience of a high volume tertiary cardiology centre. Int J Cardiovasc Imaging.

[26] Hoffmann U, Globits S, Schima W (2003). Usefulness of magnetic resonance imaging of cardiac and paracardiac masses. Am J Cardiol.

[27] Malaret GE, Aliaga P (1968). Metastatic disease to the heart. Cancer.

[28] Fujio H, Otsuki N, Horichi Y (2019). Cardiac metastasis in a living patient with oral cancer. Auris Nasus Larynx.

[29] Mousavi N, Cheezum MK, Aghayev A (2019). Assessment of cardiac masses by cardiac magnetic resonance imaging: histological correlation and clinical outcomes. J Am Heart Assoc.

